# Combined plasma and tissue genotyping of EGFR T790M benefits NSCLC patients: a real‐world clinical example

**DOI:** 10.1002/1878-0261.12481

**Published:** 2019-04-10

**Authors:** Bing Wei, Chengzhi Zhao, Jun Li, Jiuzhou Zhao, Pengfei Ren, Ke Yang, Chi Yan, Rui Sun, Jie Ma, Yongjun Guo

**Affiliations:** ^1^ Henan Cancer Hospital The Affiliated Cancer Hospital of Zhengzhou University China

**Keywords:** EGFR, liquid biopsy, NSCLC, osimertinib, PFS, T790M

## Abstract

Acquired resistance to epidermal growth factor receptor (EGFR)‐tyrosine kinase inhibitors (TKIs) is a prevalent clinical problem in the management of advanced non‐small‐cell lung cancer (NSCLC) with TKI‐sensitizing mutations in the EGFR gene. Third‐generation EGFR‐TKIs have demonstrated potent activity against TKI resistance mediated by the EGFR T790M mutation, and standard rebiopsy and liquid biopsy are utilized to assess the T790M status of the NSCLC patients who experienced progressive disease (PD). Here, we conducted a retrospective study to assess 375 patients whose initial biopsy indicated a TKI‐sensitizing mutation (either EGFR 19del or L858R) and who developed PD after treatment with first‐generation TKIs, and assayed for T790M status. We adopted a combination approach in which tissue rebiopsy is preferred, utilizing liquid biopsies when tissue rebiopsy is not feasible. We analyzed the potential predictive clinical factors affecting T790M detection, evaluated the standard rebiopsy and liquid biopsy methods in T790M genotyping, and reported the clinical performance of osimertinib. Our results suggested that primary EGFR 19del, brain metastasis, and longer progression‐free survival of initial EGFR‐TKI treatment are associated with acquired T790M resistance. T790M‐positive patients significantly benefited from osimertinib. In conclusion, the real‐world clinical adoption of the combination approach with both tissue rebiopsy and liquid biopsy for T790M genotyping may provide significant benefits to patients who have developed PD after first‐generation TKI treatments.

**Abbreviation**
ARMS‐PCR: amplification refractory mutation system PCR
DCR: disease control rate
ddPCR: digital droplet PCR
EGFR: epidermal growth factor receptor
FFPE: formalin‐fixed, paraffin‐embedded
NGS: next‐generation sequencing
NSCLC: non‐small‐cell lung cancer
NTC: nontemplate control
ORR: objective response rate
PD: progressive disease
PFS: progression‐free survival
PR: partial response
QC: quality control
TKI: tyrosine kinase inhibitors

## 1 Introduction

Lung cancer is the most common cancer and the leading cause of cancer death worldwide (Siegel *et al*., 2018). In the recent two decades, treatment of non‐small‐cell lung cancer (NSCLC), which accounts for 85% of lung cancer patients, has evolved to a great extent (Lamb and Scott, 2017; Lin *et al*., 2014; Siegel *et al*., 2018; Wu and Shih, 2018) . Epidermal growth factor receptor tyrosine kinase inhibitors (EGFR‐TKIs) provide favorable treatment options for patients who carry EGFR‐activating mutations, which most commonly appear in exon 19 (19del) and in exon 21 (L858R) (Deng *et al*., 2016; Ettinger *et al*., 2013). However, patients with advanced NSCLC harboring EGFR‐activating mutations develop progressive disease (PD) in a median response period of 10–12 months after taking first‐generation TKIs, such as erlotinib and gefitinib (Lin *et al*., 2014; O'Kane *et al*., 2018; Tan *et al*., 2018; Wu and Shih, 2018). The most common mechanism of drug resistance is associated with acquired T790M mutation (O'Kane *et al*., 2018; Tan *et al*., 2018; Wu and Shih, 2018). Recently, third‐generation EGFR‐TKIs have been reported to produce promising responses in T790M‐positive NSCLC patients. Osimertinib is approved in nearly 70 countries around the world as the first‐line treatment for patients with locally advanced or metastatic EGFR T790M mutation‐positive NSCLC whose disease has progressed during or after EGFR‐TKI therapy, with objective response rate (ORR) and disease control rate (DCR) approximately at 60% and 90% (Lamb and Scott, 2017; Minari *et al*., 2016; Stinchcombe, 2016; Tan *et al*., 2018; Yang *et al*., 2017; ). Therefore, detecting T790M mutation status is important for facilitating the application of third‐generation EGFR‐TKIs on advanced NSCLC patients who underwent PD after previous TKI treatments.

Molecular diagnosis with tissue specimens is considered the gold standard for EGFR mutation examination. However, for patients who have received TKI treatment and developed PD, it is a clinical challenge to collect tumor tissue for genotyping, as the invasive and time‐intensive procedure may be risky to these late‐stage patients and thereby infeasible, and may also delay subsequent treatment. EGFR genotyping with plasma‐circulating DNA has shown promise in terms of minimal invasiveness, accessibility, quick turnaround time, convenience, and potential clinical utility. Several different techniques were developed to examine liquid biopsy, including amplification refractory mutation system PCR (ARMS‐PCR), next‐generation sequencing (NGS), digital droplet PCR (ddPCR), BEAMing PCR (Chougule and Basak, 2017; Dearden *et al*., 2017; Huang *et al*., 2017; Jenkins *et al*., 2017; Wang *et al*., 2017). Despite its convenience, the assay's high false positive rate, which is > 20%, limits its widespread utilization compared to well‐established molecular assays on tissue biopsies, which have been systematically investigated in clinical trials. Liquid biopsy assays have been established as an alternative approach for EGFR detection in Europe when tissue biopsy is not feasible (Chougule and Basak, 2017; Dearden *et al*., 2017; Huang *et al*., 2017; Kim *et al*., 2018; Oxnard *et al*., 2016). To balance the convenience of plasma genotyping against false negative rate, an alternative paradigm has also been proposed: T790M‐positive patients can be screened by plasma genotyping, and T790M‐negative patients can undergo tissue rebiopsy to further identify T790M mutational status (Chougule and Basak, 2017; Huang *et al*., 2017; Oxnard *et al*., 2016). In the current report, we present our real‐world practice with both tissue and plasma genotyping of EGFR T790M, adopting the first paradigm in which tissue rebiopsy feasibility was the primary criteria for assay selection.

Since January 2016, our institution has adopted a digital PCR‐based EGFR T790M assay for patients developing PD after first‐generation TKI treatment and liquid biopsy was recommended when tissue rebiopsy was not clinically feasible. In the current study, we have retrospectively reviewed the new practice, investigated the association between clinical factors and T790M mutation status, and reported the clinical outcomes upon osimertinib treatment.

## 2 Materials and methods

### 2.1 Patients demographics

Our study cohort consisted of patients with advanced or recurrent NSCLC whose initial biopsy indicated an EGFR‐activating mutation (either EGFR 19del or L858R), treated with first‐generation EGFR‐TKIs (gefitinib and erlotinib), developed PD, and underwent rebiopsy or liquid biopsy at our institution (Henan Cancer Hospital, Zhengzhou, Henan, China) between January 2016 and October 2017. We retrospectively reviewed the patient's molecular testing results and clinical characteristics. Clinical efficacy outcomes were determined by image testing with CT, MRI, chest X‐ray, or PET/CT on the basis of Response Evaluation Criteria in Solid Tumors. Collection and the use of tumor and blood samples of patients in this study were in accordance with the standards required by the Declaration of Helsinki and approved by the Ethical and Institutional Review Board of Henan Cancer Hospital. All patients enrolled in this study have signed informed consents prior the initiation of the experiments.

### 2.2 Tumor specimen collection and processing

Tumor samples were collected from NSCLC patients during surgery or biopsy and processed by standard methods to make formalin‐fixed, paraffin‐embedded (FFPE) samples. DNAs from FFPE specimens were extracted using a QIAamp® DNA FFPE Tissue Kit (Qiagen, Hilden, Germany), resulting in final concentrations between 20–50 ng·μL^−1^ and OD260/280 ratio of 1.8–2.0. Cytology samples including pleural effusion were frozen, stored at −80 °C, and processed by standard methods using QIAamp® DNA Micro Kit (Qiagen).

### 2.3 EGFR mutation analyses of tissue DNA

Epidermal growth factor receptor mutations in FFPE DNA samples were detected using either ARMS real‐time PCR or NGS. For ARMS real‐time PCR, the Human EGFR Gene Mutation Detection Kit (ACCB Biotech, Beijing, China), which entails ARMS real‐time PCR of 45 mutations in exons 18–21 of the EGFR gene by mutant‐specific primers, was used. The assay was sensitive enough to detect as low as 1% in 10 ng·μL^−1^ of genomic DNA. Quantitative PCR was performed on an Agilent Mx3000P system (Agilent, Technologies Santa Clara, CA, USA).

### 2.4 Plasma collection and ctDNA extraction

For ctDNA extraction, a total amount of 10–15 mL whole blood was specifically collected into EDTA tubes for routine test of T790M assay by ddPCR assay accompanied with NGS validation. Five milliliters of plasma was separated within 1 h after blood collection. Briefly, whole blood at room temperature was centrifuged at 1600 ***g*** for 20 min, and the supernatant was transferred to a new tube and centrifuged at 1600 ***g*** for 5 min. Plasma was then transferred to a new 2‐mL tube, and the plasma ctDNAs were extracted using the Plasma Circulating Nucleic Acid Preparation Kit (Qiagen), following the manufacturer's instructions. Extracted ctDNAs were then used directly in EGFR mutation analyses. The quality and quantity of ctDNA were evaluated by Qubit (Qubit™ dsDNA HS Assay Kit, Life Technologies, Waltham, MA,USA) and Agilent 2100 Bioanalyzer System (High Sensitivity DNA Chips, Agilent Technologies). For NGS, the starting volume was 10 mL.

### 2.5 Droplet digital PCR assay for EGFR T790M detection in plasma ctDNA

Digital droplet PCR was performed using QX200 Droplet Digital PCR system (Bio‐Rad, Hercules, CA, USA). The final volume of the PCR mixture was 20 μL with PrimePCR Mutation Assay (EGFR T790M; Bio‐Rad, #1863103) for detecting ctDNA in patient plasma containing 10 μL of ddPCR Supermix for Probes (No dUTP), 1 μm of each primer, 0.25 μm of each probe, 200 μm of dNTP, and 6 μL of DNA (from ~ 2 mL of blood sample or control DNA), with or without 5 μm of blocking oligo. The following PCR conditions for ddPCR were used: (a) an initial denaturation step at 95 °C for 10 min followed by (b) 40 cycles at 94 °C for 15 s and 58 °C for 60 s and (c) 40 cycles at 98 °C for 10 min and 4 °C for 4 min. Each ramp rate was smaller or equal to 2 °C per second. PCR products were then subjected to analysis by the QX‐200 droplet reader and quantasoft analysis software (Bio‐Rad). Both nontemplate control (NTC) and wild‐type control were utilized as quality control (QC) of the assay. Wild‐type control had ‘ch1+’ ≤ 3 droplets within the range. In addition, deionized distilled water was used as negative control (NTC) with no positive droplets identified. The criteria for determining the positive results are defined as ‘ch1+’ > 3 droplets in detected samples.

### 2.6 Next‐generation sequencing with tissue rebiopsy

Library preparation was performed following manufacturer's protocol (Cat#RS0103F‐12; Burning Rock Biotech, Guangzhou, China). Briefly, DNA shearing was performed followed by end repair, phosphorylation, adaptor ligation, and purification using Agencourt AMPure beads (Beckman Coulter, Fullerton, CA, USA). DNA fragments were captured with probe baits and selected with magnetic beads followed by subsequent PCR amplification. Indexed samples were sequenced on a MiSeq system (Beckman Coulter) with paired‐end reads. The QC criteria are as follows: The initial input of extracted DNA should be contained in the range of 30–200 ng. Qubit was used for quantitative measurement and QC of the DNA template. DNA fragment length should reach 200–250 bp after shearing by ultrasonic wave. The average sequencing depth should be ≥ 2000× with the clear reads > 300×. Q30 should be > 90%.

### 2.7 Next‐generation sequencing with plasma ctDNA

Library construction was performed following manufacturer's protocol (#EP0301708, ANNOROAD Gene Technology, Beijing, China) for end repair, adapter ligation, and purification by Agencourt AMPure beads (Beckman Coulter) as previously described (Yang *et al*., 2018). DNA fragments should be captured with probes followed by subsequent PCR amplification. DNA samples were subsequently sequenced by NextSeq 550AR (Illumina, San Diego, CA, USA). The QC criteria of the NGS assay with liquid biopsies included the following: initial DNA input 10–100 ng by Qubit (QubitTM dsDNA HS Assay Kit, Life Technologies); DNA fragments range from 160 to 180 bp with major peak at 170 bp; average sequencing depth is > 20 000×; clear reads should be > 4000×; and Q30 is ≥ 95%.

### 2.8 Validation of discordant results using NGS

When plasma T790M genotyping (ddPCR) was discordant from tumor T790M genotyping (ARMS‐PCR) from identical patients, repeated genotyping was performed by using NGS on samples with positive results. The NGS methodology applied can be referred to the previous description.

### 2.9 Statistical analyses

Statistical analysis was performed using spss Statistics 19 software (IBM SPSS Statistics, Armonk, NY, USA). Univariate analyses were conducted with the chi‐squared test to evaluate differences in categorical variables between T790M‐positive and T790M‐negative groups. Survival curves were derived using the Kaplan–Meier method and compared using the log‐rank test. Progression‐free survival (PFS) was defined as the period from the date of initiation of first‐line EGFR‐TKI treatment to the date of disease progression. *P* values < 0.05 were considered statistically significant.

## 3 Results

### 3.1 Patient population

**Figure 1:**
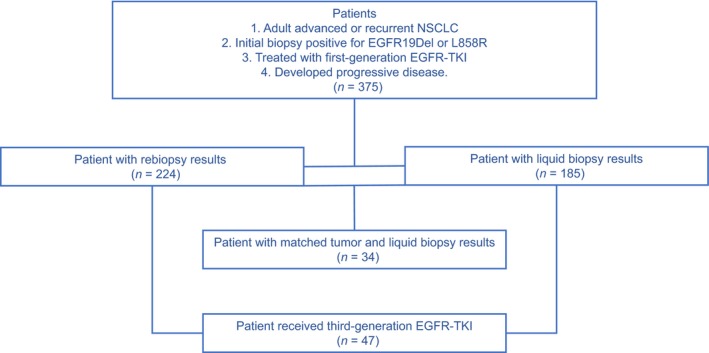
Flow diagram of patient population. Out of 375 patients eligible for the study, 224 received T790M genotyping with tissue rebiopsy, 185 with liquid biopsy, and 34 with both. A total of 47 patients received third‐generation EGFR‐TKI.

We retrospectively reviewed all the patients who had advanced or recurrent NSCLC, had initial biopsy positive for TKI sensitive mutations (either *EGFR* exon 19 deletion, or *EGFR* L858R), received first‐generation TKI treatment, developed PD, and were tested for EGFR T790M upon progression in our institution from January 2016 to September 2017. A total of 375 patients were eligible and included for the study (Fig. 1), and the demographics are shown in Table 1. The median age was 59 years old (range: 27–92). The percentage of males, nonsmokers, and patients with adenocarcinoma were 34.9% (131/375), 73.9% (277/375), and 98.9% (371/375), respectively (Table 1).

**Table 1:** Patient information and comparison of T790M mutation status

	Total population (*n* = 375)	T790M positive (*n* = 194)	T790M negative (*n* = 181)	*P*‐value
Age (at rebiopsy)				
< 60 years	198	97	101	0.261
≥ 60 years	177	97	80	
Sex				
Male	131	62	69	0.211
Female	244	132	112	
Smoking status				
Smoker	97	46	51	0.338
Nonsmoker	277	147	130	
Brian metastasis				
With	188	107	81	0.034*
Without	183	84	99	
Initial EGFR mutation				
Exon 19 deletion	217	122	95	0.042*
L858R	158	72	86	
Initial EGFR‐TKI				
Erlotinib	54	31	23	0.367
Gefitinib	321	163	158	
PFS of initial EGFR‐TKI				
< 13 months	225	103	122	0.005**
≥ 13 months	150	91	59	
Biopsy methods#				
Rebiopsy	224	119	105	0.312
Liquid biopsy	185	89	96	

*P‐value < 0.05; **P‐value < 0.005.

Prior to EGFR‐TKI treatment, 217 patients had been diagnosed carrying EGFR 19del mutation (57.9%), while 158 had EGFR L858R mutation (42.1%) detected from their initial tissue biopsy. The two types of major mutations were mutually exclusive. Upon PD, all patients received EGFR T790M testing, and liquid biopsy was recommended when tissue rebiopsy was not clinically feasible. In total, 224 patients underwent rebiopsy for tissue genotyping of T790M, while 185 underwent liquid biopsy for circulating tumor DNA testing. There were only 34 patients who had both tissue rebiopsy and plasma samples obtained for T790M mutation analysis (Fig. 1). Forty‐seven patients received third‐generation EGFR‐TKI (osimertinib) treatment with follow‐ups till March 2018.

### 3.2 Evaluation of liquid biopsy‐based T790M assay

Before adoption in the clinic, the digital PCR‐based T790M assay had been evaluated using archived tissue biopsies and matched blood samples, achieving sensitivity and specificity of 76.7% and 100% (data not shown). In the current study, we reviewed 34 cases that were tested with both liquid biopsy and rebiopsy samples to review real‐world performance using the same concordance methodology. In these 34 patients, the T790M‐positive rate was 22/34 (64.7%) in tumor rebiopsy samples and 16/34 (47.1%) in ctDNA samples. The sensitivity and specificity of plasma ddPCR assay were 63.64% and 83.3%, respectively (Table 2). The overall positive rate for the all samples tested with tissue rebiopsy and liquid biopsy was 53.1% (119/224) and 48.1% (89/185) with no statistical significance (*P *=* *0.312).

**Table 2:** Comparison of T790M mutation status in matched liquid biopsy and rebiopsy samples

	Rebiopsy sample		Total
	T790M+	T790M−	
Plasma ctDNA			
T790M+	14	2	16
T790M−	8	10	20
Total	22	12	34
Sensitivity	63.6%		
Specificity	83.3%		

We further examined all 10 discordant samples with an independent NGS assay for the eight samples that were liquid biopsy negative/rebiopsy positive. We utilized remaining DNA extracted from tissue samples, and the NGS results were consistent with original PCR genotyping. For the two samples that were positive for T790M in plasma but negative in tissue, liquid biopsy was validated by NGS for confirming the positivity of T790M. Therefore, the ten patients were confirmed as positive for EGFR T790M. The discordance of the paired FFPE and liquid biopsy samples of the same patients could be attributed to the differences in the collection locations between the primary sites and metastatic liver and lymphoid tissue.

It is noteworthy that two patients who were positive for T790M in liquid biopsy and negative for T790M in tissue biopsy with discordant diagnostic results were treated with osimertinib. One patient did not respond to the treatment and the other one showed PD after 4 months. Both patients changed to chemotherapy after the failure of treatment by osimertinib.

### 3.3 Initial *EGFR* 19del and brain metastasis associated with *EGFR* T790M mutation

The association between various baseline clinical factors and status of *EGFR* T790M was analyzed with all 375 patients pooled as one cohort. The ten discordant samples were regarded as T790M positive based on NGS assay results. Out of 375 patients, 194 were positive for T790M (51.7%). There were no significant differences in age at rebiopsy, sex, smoking history, or the first‐generation TKI used (erlotinib or gefitinib) in relation to the presence or absence of T790M (Table 1).

The EGFR exon 19 deletion was shown to be significantly associated with T790M mutation (*P *=* *0.042). Patients who harbored *EGFR* 19del showed a significantly higher frequency (56.2%, 122/217) of developing T790M mutation than those harbored *EGFR* L858R (45.6%, 72/158; Table 1). At the time of biopsy, the existence of brain metastasis was positively correlated with T790M mutation (*P* = 0.034) with a mutation frequency of 56.9% (107/188; Table 1).

### 3.4 Progression‐free survival of initial EGFR‐TKI treatment associated with *EGFR* T790M mutation

**Figure 2:**
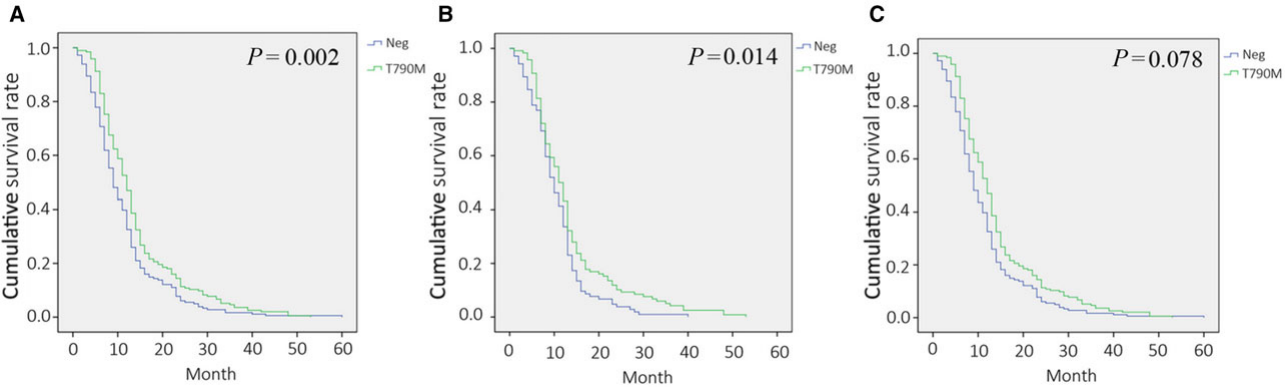
Efficacy of initial EGFR‐TKI treatment in relation to presence of T790M (PFS1). Kaplan–Meier Survival curves in (A) all patients, (B) patients with rebiopsy samples, and (C) patients with liquid biopsy samples. Curves represent T790M groups are green, and T790 wild‐type groups are blue.

The PFS of initial EGFR‐TKI treatment (PFS1) was evaluated by different EGFR T790M mutation status in three scenarios: all patients (Fig. 2A); patients whose T790M status was tested with rebiopsy samples (Fig. 2B); and patients with liquid biopsy (Fig. 2C). In the all‐patient scenario, the mean PFS1 was 11.7 months (95% CI: 10.0–12.5 months). The median PFS1 was longer in the T790M positive group (14.1 months, 95% CI: 12.8–15.5 months) than in the negative group (11.2 months, 95% CI: 10.0–12.5 months; *P* =* *0.002; Fig. 2A). Patients who received EGFR‐TKI treatment for at least 13 months showed a higher frequency of T790M (60.7%, 91/150) than those who did not (45.8%, 103/235; *P *=* *0.005; Table 1 and Fig. 2A). Consistently, in patients whose T790M status was tested with rebiopsy tissue samples, the mean PFS1 was longer in the T790M‐positive group (13.6 months, 95% CI: 11.8–15.3 months) than in the negative group (10.6 months, 95% CI: 9.4–11.9; *P *=* *0.014; Fig. 2B). The same trend was observed in patients whose T790M status was examined with liquid biopsy; however, the difference is not statistically significant (*P *=* *0.078). The mean PFS1 in the liquid biopsy group was 13.6 months (95% CI: 11.8–15.3 months) with positive T790M and was 10.6 months (95% CI: 9.4–11.9 months) for negative group (Fig. 2C).

### 3.5 Clinical outcomes of osimertinib in T790M‐positive patients

With the limited access of osimertinib in China from 2016 to 2017, only 47 patients who acquired T790M resistant mutation received osimertinib treatment. The ORR was 66.0% (31/47 patients), and the DCR was 91.5% (43/47 patients). The PFS upon osimertinib treatment (PFS2) was 13.4 months (95% CI: 10.7–16.1 months; Table 3). The efficacy outcomes were consistent with published osimertinib studies (Dearden *et al*., 2017; Siegel *et al*., 2018). No significant difference was observed in terms of ORR, DCR, or PFS2 among patients tested with liquid biopsy or tissue rebiopsy.

**Table 3:** Efficacy outcomes in current study compared with selected osimertinib clinical trials

Study	ORR (%)	DCR (%)	PFS (month)
Current study	66	91	13.6
AURA	61	95	9.6
AURA extension	62	90	12.3
AURA 2	70	92	9.9

**Figure 3:**
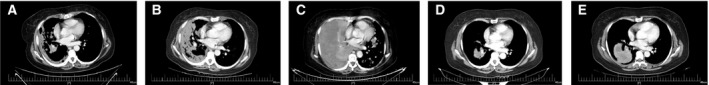
CT scans of one patient with PR to osimertinib treatment. (A) September 2015, a tumor mass of the right pulmonary hilum accompanied by mediastinal lymph node metastasis; (B) January 2016, a dramatic reduction of primary tumor and metastasis, indicating PR to initial gefitinib; (C) April 2017, large patchy high‐density shadow in the right lower lobe and new metastasis developed in both lungs, indicating PD; (D) May 2017, 1‐month follow‐up of osimertinib treatment, and (E), November 2017, 7‐month follow‐up, dramatic reduction of shadows in the right lung and metastasis in the left lung, indicating PR to osimertinib treatment.

We represented one case with partial response (PR) upon osimertinib treatment (Fig. 3). The patient was admitted to our hospital in September 2015 with CT scan revealing a tumor mass of the right pulmonary hilum accompanied by mediastinal lymph node metastasis (Fig. 3A). As an activating *EGFR* mutation (19del) was detected with high mutation allele frequency, oral gefitinib treatment was initiated. CT scan in January 2016 demonstrated PR with a dramatic reduction of primary tumor and metastasis (Fig. 3B). At 20 months in April 2017, CT scan showed large patchy high‐density shadow in the right lower lobe and new metastasis development in both lungs, indicating PD (Fig. 3C). EGFR genotyping with rebiopsy showed acquired T790M, revealing the acquired resistance mechanism. The patient was then accepted to participate the clinical trial of AZD9291 to receive osimertinib treatment. In the 1‐ and 7‐month follow‐ups, CT scan showed dramatic reduction of shadows in the right lung and metastasis in the left lung (Fig. 3D,E), resulting in PR.

We identified four patients who developed C797S‐resistant mutation upon osimertinib treatment by NGS liquid biopsy assays (Table 4). Out of the four patients, three harbored C797S in *cis* with T790M and one in trans. The resistance to osimertinib was observed at 23, 23, 22, 10 months, respectively. It is noteworthy that the latter one also harbored MET amplification, and PFS2 was relatively shorter (10 months).

**Table 4:** Clinical histories of four patients harboring dual mutations of T790M and C797S

Patient	#1	#2	#3	#4
First biopsy	19Del	L858R	19Del	L858R
Inpatient ID	151794	359089	317038	358463
First‐generation TKI treatment	Gefitinib	Gefitinib	Gefitinib	Gefitinib
Treatment duration (months)	69	22	47	29
Rebiopsy	19Del + T790M	L858R + T790M	19Del + T790M	L858R + T790M
Osimertinib treatment (months)	23	23	22	10
Third biopsy (liquid biopsy)	19Del + T790M + C797S Cis	L858R + T790M + C797S Cis	19Del + T790M + C797S Cis	L858R + T790M + C797S Trans + Met Amplification
Subsequent Treatment	Chemotherapy + radiotherapy	Chemotherapy	Apatinib	Chemotherapy

## 4 Discussion

The genotyping of *EGFR* T790M has gradually been established as routine molecular testing for patients relapsing after treatment of the first‐ or second‐generation EGFR‐TKIs, as T790M‐positive patients may achieve promising clinical outcomes from osimertinib (Dearden *et al*., 2017; Lamb and Scott, 2017; Oxnard *et al*., 2016; Stinchcombe, 2016). In order to understand the resistance mechanism and evaluate the clinical utility of T790M testing, we investigated a total of 375 patients who harbored one of two *EGFR*‐activating mutations (19del or L858R), relapsing from the first‐generation TKI treatment, and were tested with *EGFR* T790M assays in real‐world clinical settings.

T790M represents ~ 60% of first‐ and second‐generation TKI resistance mechanism; MET amplification, HER2 amplification, PIK3CA mutation, and BRAF mutations collectively account for ~ 20%; histological transformation from NSCLC to small cell lung cancer accounts for ~ 5%; the remaining mechanisms remain unknown (Faehling *et al*., 2017; Lim *et al*., 2018; Lin *et al*., 2014). KRAS has been associated in 9% of cases with resistance to first/second generations TKI (Re *et al*., 2017). By pooling all non‐T790M patients together, we showed that PFS after initial TKI treatment (PFS1) was significantly higher in T790M than non‐T790M patients (14.1 months vs 11.2 month, *P *=* *0.002, Fig. 2), which is consistent with several previous reports (Kuiper *et al*., 2014; Matsuo *et al*., 2016; Wang *et al*., 2017, 2018). The difference of PFS1 might be due to the complexed underlying resistance mechanisms: On one hand, T790M‐positive cells are selected and undergo enrichment after long‐term exposure to TKIs (Kawamura *et al*., 2018; Wang *et al*., 2017, 2018), while on the other hand, the non‐T790M patients harbored some resistance mechanisms, such as KRAS mutation and/or unknown mechanisms, which may contribute to a worse TKI response compared with T790M.

Many groups have explored whether initial *EGFR*‐activating mutation subtypes might serve as a prognostic factor for TKI treatment and possible TKI resistance with T790M mutation (Deng *et al*., 2016; Ma *et al*., 2017; Matsuo *et al*., 2016; O'Kane *et al*., 2018; Zhuo *et al*., 2017). Recently, a meta‐analysis by Wu group suggested that tumors harboring *EGFR* 19del had a higher T790M mutation rate than those having L858R (Deng *et al*., 2016). Our data are consistent with the meta‐analysis, showing a significant difference (*P *=* *0.042). We speculate that the difference is relatively subtle and thereby might be easily compromised in cohorts with relatively small patient numbers as in many studies reporting no significant difference. It has been hypothesized that cells harboring *EGFR*19del and L858R mutations employ different molecular mechanisms to respond to TKI treatment and develop resistance, which will need rigorous investigation.

Our institution has adopted the scheme that T790M genotyping with tissue rebiopsy is preferred, utilizing liquid biopsies when tissue rebiopsy is not feasible. The major reasons for choosing this over the liquid biopsy screening paradigm were the vast clinical information available from tissue rebiopsy in addition to T790M status and the reduction of sequential and repeated molecular testing with this workflow. With the current cohort of 375 patients, we retrospectively evaluated the workflow. On one hand, tissue rebiopsy could produce more reliable results than liquid biopsy. Firstly, the analytical sensitivity remains under 80%, potentially leading to less optimized treatment decision for patients who would otherwise benefit from osimertinib. In addition, although both tissue rebiopsy and liquid biopsy subgroups exhibit similar trends that T790M patients had longer PFS1, only the tissue rebiopsy subgroup showed statistical significance (Fig. 1). On the other hand, liquid biopsy provides valuable opportunities for a large group of patients to get access to better treatment option. It was not feasible to obtain tissue rebiopsy for 151 patients, which was 40.2% of all patients. In addition, high specificity of liquid biopsy is well recognized. In fact, the specificity was 100% in the current study, after the two ddPCR‐positive‐tissue‐negative samples were confirmed as positive by NGS assay. Our result supports the clinical utility of the combination strategy in the real world, as the overall T790M‐positive rate, and ORR and DCR for osimertinib, were all consistent with data published with clinical trials.

## 5 Conclusion

Overall, our data strongly support that a combination of liquid biopsy and tissue rebiopsy for T790M genotyping could benefit patients who have developed PD after first‐generation TKI treatments in real‐world clinical settings. In addition, the combined utilization of ARMS‐PCR, ddPCR, and NGS are recommended for precise diagnosis with maximum validation and optimal selection of patients who may benefit from TKI treatment.

## Conflict of interest

The authors declare no conflict of interest.

## Author contributions

WB conceived the experiment design. WB and CZ performed most of the experiments and wrote the manuscript. JL and JZ conducted the ddPCR experiments. PR and KY conducted statistic analyses. CY and RS participated in sample and data collection process. JM and YG supervised the study and revised the paper.
